# Roles of the Raf/MEK/ERK and PI3K/PTEN/Akt/mTOR pathways in controlling growth and sensitivity to therapy-implications for cancer and aging

**DOI:** 10.18632/aging.100296

**Published:** 2011-03-10

**Authors:** Linda S. Steelman, William H. Chappell, Stephen L. Abrams, C. Ruth Kempf, Jacquelyn Long, Piotr Laidler, Sanja Mijatovic, Danijela Maksimovic-Ivanic, Franca Stivala, Maria C. Mazzarino, Marco Donia, Paolo Fagone, Graziella Malaponte, Ferdinando Nicoletti, Massimo Libra, Michele Milella, Agostino Tafuri, Antonio Bonati, Jörg Bäsecke, Lucio Cocco, Camilla Evangelisti, Alberto M. Martelli, Giuseppe Montalto, Melchiorre Cervello, James A. McCubrey

**Affiliations:** ^1^Department of Microbiology and Immunology, East Carolina University, Greenville, NC 27858, USA; ^2^Department of Physics, East Carolina University, Greenville, NC 27858, USA; ^3^Department of Medical Biochemistry, Jagiellonian University Medical College, Krakow, Poland; ^4^Department of Immunology, Instititue for Biological Research “Sinisa Stankovic”, University of Belgrade, Belgrade, Serbia; ^5^Department of Biomedical Sciences, University of Catania, Catania, Italy; ^6^Regina Elena Cancer Center, Rome 00144, Italy; ^7^University of Rome, La Sapienza, Department of Hematology-Oncology, Rome 99161, Italy; ^8^University Hospital of Parma, Unit of Hematology and Bone-Marrow Transplantation, Via Gramsi n.14, Parma 43100, Italy; ^9^Department of Medicine, University of Göttingen, Göttingen, Germany; ^10^Dipartimento di Scienze Anatomiche Umane e Fisiopatologia dell'Apparato Locomotore, Università di Bologna, Bologna, Italy; ^11^IGM-CNR, Sezione di Bologna, C/o IOR, Bologna, Italy; ^12^Department of Internal Medicine and Specialties, University of Palermo, Palermo, Italy; ^13^Consiglio Nazionale delle Ricerche, Istituto di Biomedicina e Immunologia, Molecolare “Alberto Monroy”, Palermo, Italy

**Keywords:** Raf, MEK, PI3K, mTOR, cancer, kinases, protein phosphorylation, signal transduction, apoptosis

## Abstract

Dysregulated signaling through the Ras/Raf/MEK/ERK and PI3K/PTEN/Akt/mTOR pathways is often the result of genetic alterations in critical components in these pathways or upstream activators. Unrestricted cellular proliferation and decreased sensitivity to apoptotic-inducing agents are typically associated with activation of these pro-survival pathways. This review discusses the functions these pathways have in normal and neoplastic tissue growth and how they contribute to resistance to apoptotic stimuli. Crosstalk and commonly identified mutations that occur within these pathways that contribute to abnormal activation and cancer growth will also be addressed. Finally the recently described roles of these pathways in cancer stem cells, cellular senescence and aging will be evaluated. Controlling the expression of these pathways could ameliorate human health.

## INTRODUCTION

The Ras/Raf/MEK/ERK and Ras/PI3K/PTEN/Akt/mTOR signaling pathways have been shown over the past 25 years to play key roles in the transmission of proliferative signals from membrane bound receptors. Mutations can occur in the genes encoding pathway constituents (*e.g*., *RAS*, *RAF*, *PIK3CA*, *PTEN*, *AKT*, *TSC1*, *TSC2*) or in upstream receptors which activate these pathways. These pathways relay this information through interactions with various other proteins to the nucleus to control gene expression [1-13]. This review will discuss how these pathways may be aberrantly regulated by either upstream mutations/amplification or by intrinsic mutations of key components of these signaling pathways. Elevated levels of activated components of these pathways are often associated with poor prognosis in cancer patients or premature aging [2-5, 7]. Increased expression of signaling pathways can also be correlated with altered sensitivity to targeted therapy compared to patients that do not exhibit elevated expression [2-4]. Inhibition of Raf, MEK, PI3K, Akt and mTOR may prove useful in cancer treatment as well as in preventing or suppressing cellular aging. These observations have propelled the pharmaceutical industry to develop inhibitors that target key components of these pathways.

The Ras/Raf/MEK/ERK and Ras/PI3K/PTEN/Akt/mTOR signaling pathways consist of kinases cascades that are regulated by phosphorylation and de-phosphorylation by specific kinases, phosphatases as well as GTP/GDP exchange proteins, adaptor proteins and scaffolding proteins. The regulation of these cascades can be much like the axiom of real estate, “location-location-location”, as the membrane localization of these components is often critical for their activity, even though some members of these pathways can function in other cellular regions (*e.g.,* mitochondrion, nucleus). Indeed, one emerging observation in both extracellular signal-regulated kinase 1 and 2 (ERK1/2) and mammalian target of rapamycin (mTOR) signaling is the realization that pathways generate specific biological responses dependent upon where in the cell the signal originates [12]. For example, phosphorylation of both epidermal growth factor receptor (EGFR) and cytosolic phospholipase A(2) [cPLA(2)] is most prominent when ERK1/2 is activated from lipid rafts, whereas p90 Ribosomal S6 kinase-1 (p90^Rsk-1^) is mainly activated by Ras signals emanating from disordered membranes. This substrate selectivity is governed by the participation of different scaffold proteins that distinctively couple ERK1/2, activated at defined subcellular domains, to specific substrates. Ras subcellular localization can determine substrate specificity through distinct utilization of scaffold proteins [1, 6, 12]. Clearly the subcellular localization of pathway components and the presence of various adaptor and scaffolding molecules are critical for the activity of these pathways. The regulation and function of these two pathways will be concisely reviewed as well as the effects of genetic mutations that are important in human cancer.

### The Ras/Raf/MEK/ERK Pathway

**Figure 1.**
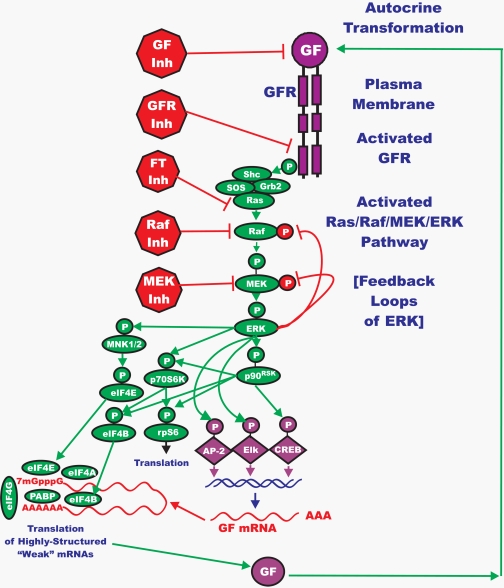
Overview of the Ras/Raf/MEK/ERK Pathway and Potential Sites of Therapeutic Intervention with Small Molecule Membrane-Permeable Inhibitors The Ras/Raf/MEK/ERK pathway is regulated by Ras (indicated in green ovals), as well as various upstream growth factor receptors (indicated in purple) and non-receptor kinases. Sites where various small molecule inhibitors suppress this pathway are indicated by red octagons. The downstream transcription factors regulated by this pathway are indicated in purple diamond shaped outlines. The Ras/Raf/MEK/ERK pathway also interacts with key proteins involved in protein translation (indicated in green ovals). The Ras/Raf/MEK/ERK pathway aids in the assembly of the protein translation complex responsible for the translation of “weak” mRNAs (indicated in a red line folding over on itself) important in the prevention of apoptosis. This drawing depicts a relative common, yet frequently overlooked phenomenon in human cancer, autocrine transformation. GF = growth factor, GFR = growth factor receptor.

An introductory overview of the Ras/Raf/MEK/ERK pathway is presented in Figure 1. Also outlined in this figure are common sites of intervention with signal transduction inhibitors. Many of these inhibitors have been evaluated in various clinical trials and some are currently being used to treat patients with specific cancers. Extensive reviews of many inhibitors targeting these pathways have been recently published [2-4]. This figure serves as a starting reference point for understanding the flow of information through the Ras/Raf/MEK/ERK pathway from a growth factor to a specific receptor to phosphorylation of appropriate transcription factors in the nucleus, which modulate the expression of key genes [7-11]. The effects of this pathway on the translational apparatus are also diagrammed. Often mRNAs encoding growth factors are entitled “weak” mRNAs and require the effects of the Ras/Raf/MEK/ERK and Ras/PI3K/PTEN/Akt/mTOR pathways for efficient translation [2, 4, 11]. As an example, we present the autocrine production of a growth factor. Importantly, many components and interacting members of this pathway are also present as mutated forms in the genomes of retroviruses that induced cancer in experimental animals. Thus there have always been direct pivotal links of this pathway with malignancy.

After growth factor/cytokine/mitogen stimulation of the appropriate (cognate) receptor, a Src homology 2 domain containing protein (Shc) adaptor protein becomes associated with the C-terminus of the specific activated growth factor receptor (*e.g*., vascular endothelialgrowth factor receptor [VEGFR], epidermal growth factor receptor [EGFR], insulin like growth factor-1 receptor [IGF-1R] and many others) [2-4]. Shc recruits the Grb2 protein and the son of sevenless (SOS) homolog protein, resulting in the loading of membrane-bound Ras with GTP [7]. Ras can also be activated by growth factor receptor tyrosine kinases [GFRTK], such as insulin receptor (IR), via intermediates like insulin receptor substrate (IRS) proteins that bind growth factorreceptor-bound protein 2[7, 8]. Ras:GTP then recruits Raf to the membrane where it becomes activated, likely via a Src-family tyrosine (Y) kinase [9]. At this point we will be somewhat generic, although it should be pointed out that both Ras and Raf are members of multigene families and there are three Ras members (Ki-Ras, N-Ras and Ha-Ras) [7] and three Raf members (B-Raf, Raf-1 [*a.k.a* c-Raf] and A-Raf) [9]. Raf is responsible for serine/threonine (S/T) phosphorylation of mitogen-activated protein kinase kinase-1 (MEK1)[2, 3, 7]. MEK1 phosphorylates ERK1 and 2 at specific T and Y residues [10]. Activated ERK1 and ERK2 serine S/T kinases phosphorylate and activate a variety of substrates, including p90^Rsk1^ [2, 3, 7, 10]. ERK1/2 has many downstream and even upstream substrates (see below). p90^Rsk1^ can activate the cAMP response element binding protein (CREB) transcription factor [13].

The number of ERK1/2 targets is easily in the hundreds (>600). Thus suppression of MEK and ERK activities will have profound effects on cell growth and aging. Activated ERK can also phosphorylate B-Raf, Raf-1 and MEK1 which alter their activity (Figure 1). Depending upon the site phosphorylated on Raf-1, ERK phosphorylation can either enhance [14] or inhibit [15] Raf-1 activity. In contrast, when B-Raf [16] or MEK1 [17] are phosphorylated by ERK, their activity decreases. These phosphorylation events serve to alter the stability and/or activities of the proteins. This is the first discussion of feed-back loops which will become important in consideration of whether to just target MEK or to target both Raf and MEK in various cancers. It is important that the reader realize that certain phosphorylation events can either inhibit or repress the activity of the affected protein. This often depends on the particular residue phosphorylated on the protein which can confer a different configuration to the protein or target the protein to a different subcellular localization that may result in proteasomal degradation. Furthermore, as previously mentioned, certain phosphorylation events will actually serve to shut off or slow down the pathway. Thus protein phosphorylation by the Ras/Raf/MEK/ERK pathway is a very intricate process which serves to fine tune the signal often originating from a growth factor or mitogens.

Activated ERK can translocate to the nucleus and phosphorylate additional transcription factors, such as Elk-1, CREB, Fos and globin transcription factor 1 (Gata-1) and others [2-4, 14, 18], that bind promoters of many genes, including growth factor and cytokine genes that are important in promoting growth and preventing apoptosis of multiple cell types. Under certain circumstances, aberrant regulation of this pathway can contribute to abnormal cellular proliferation which may lead to many abnormalities including; autocrine transformation, drug resistance, senescence or premature aging [2, 4, 5, 19].

### The Ras/PI3K/PTEN/Akt/mTOR Pathway

**Figure 2.**
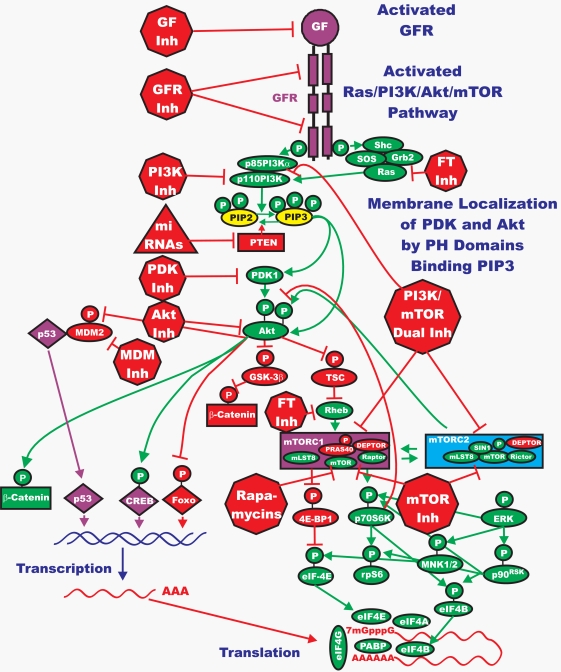
Overview of the Ras/PI3K/PTEN/Akt/mTOR Pathway and Potential Sites of Therapeutic Intervention The Ras/PI3K/PTEN/mTOR pathway is regulated by Ras (indicted in green ovals), as well as various upstream growth factor receptors (indicated in purple). Sites where various small molecule inhibitors suppress this pathway are indicated by red octagons. Naturally occurring miRNAs have been discovered to certain components of this pathway (*e.g.*, PTEN) and are indicated in a red triangle; other miRNAs to other components, especially tumor suppressor genes will likely be discovered. The downstream transcription factors regulated by this pathway are indicated in diamond shaped purple (active) or red (inactivated) outlines. This drawing depicts some of the complicated regulations of this pathway by both positive and negative phosphorylation events which serve to fine tune this pathway. Phosphorylation of some molecules by certain kinases (*e.g.*, phosphorylation of β-catenin by glycogen synthase kinase-3β [GSK-3β], indicated in red oval) results in their proteosomal degradation (indicated in red box), while phosphorylation of some molecules by certain kinases (*e.g.*, β-catenin by Akt) results in their activation (nuclear translocation, indicated in green box). The Ras/PI3K/PTEN/Akt/mTOR pathway plays a key role in regulating p53 activity (indicated in purple diamond) by phosphorylating MDM2 (indicated in red oval) which controls the stability of p53 by ubiquitination. The Ras/PI3K/PTEN/Akt/mTOR pathway plays a key role in regulating critical proteins involved in protein translation (indicated in green ovals), especially those necessary for the translation of “weak” mRNAs (mTORC1, grouped together a purple box). This pathway also indicates that Akt can result in the activation of downstream mTOR which can subsequently serve as either a negative feed back to inactivate Akt by p70S6K or activate Akt by mTORC2 (grouped together in a blue box). GF = growth factor, GFR = growth factor receptor.

An introductory overview of the Ras/PI3K/PTEN/Akt/mTOR pathway is presented in Figure 2. Also outlined in this diagram are common sites of intervention with signal transduction inhibitors. Many of these inhibitors have been evaluated in various clinical trials and some are currently being used to treat patients with specific cancers. Extensive reviews of many inhibitors targeting these pathways have been recently published [2, 4, 19, 20]. Phosphatidylinositol-3-kinase (PI3K) is a heterodimeric protein with an 85-kDa regulatory subunit and a 110-kDa catalytic subunit (*PIK3CA*) [20, 21]. PI3K serves to phosphorylate a series of membrane phospholipids including phosphatidylinositol4-phosphate (PtdIns(4)P) and phosphatidylinositol4,5-bisphosphate (PtdIns(4,5)P_2_), catalyzing the transfer of ATP-derived phosphate to the D-3 position of the inositol ring of membrane phosphoinositides, thereby forming the second messenger lipids phosphatidylinositol3,4-bisphosphate (PtdIns(3,4)P_2_) and phosphatidylinositol3,4,5-trisphosphate (PtdIns(3,4,5)P_3_) [4, 19, 20]. Most often, PI3K is activated via the binding of a ligand to its cognate receptor, whereby p85 associates with phosphorylated tyrosine residues on the receptor via a Src-homology 2 (SH2) domain. After association with the receptor, the p110 catalytic subunit then transfers phosphate groups to the aforementioned membrane phospholipids [4, 19, 20]. It is these lipids, specifically PtdIns(3,4,5)P_3_, that attract a series of kinases to the plasma membrane thereby initiating the signaling cascade [4, 19, 20].

Downstream of PI3K is the primary effector molecule of the PI3K signaling cascade, Akt/protein kinase B (PKB). Akt was originally discovered as the cellular homologue of the transforming retrovirus AKT8 and as a kinase with properties similar to protein kinases A and C [4, 19, 21, 22]. Akt contains an amino-terminal pleckstrin homology (PH) domain that serves to target the protein to the membrane for activation [21, 22]. Within its central region, Akt has a large kinase domain and is flanked on the carboxy-terminus by hydrophobic and proline-rich regions [22]. Akt is activated via phosphorylation of two residues: T308 and S473.

**Figure 3.**
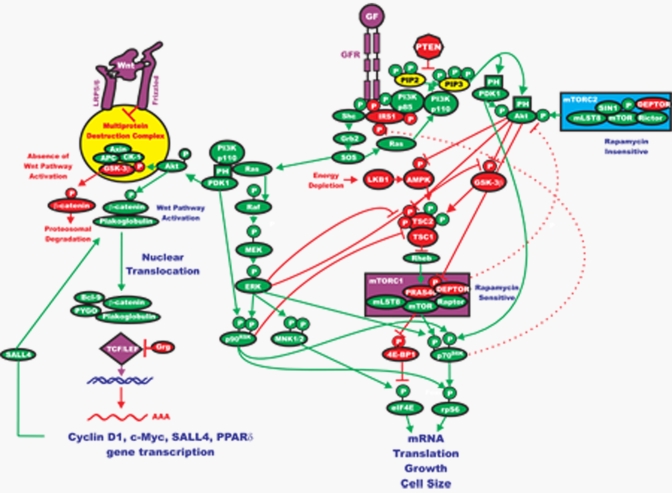
Interactions between the Ras/Raf/MEK/ERK, Ras/PI3K/PTEN/mTOR and Wnt/β-Catenin Pathways that Result in the Regulation of Protein Translation and Gene Transcription The Ras/Raf/MEK/ERK and Ras/PI3K/PTEN/Akt/mTOR pathways can affect protein translation by complex interactions regulating the mTORC1 (grouped together in a purple box) and mTORC2 (grouped together in a blue box) complexes. GF stimulation results in GFR activation which can activate both the Ras/Raf/MEK/ERK and Ras/PI3K/PTEN/Akt/mTOR pathways. Akt can phosphorylate and inhibit the effects of GSK-3β, TSC2 and PRAS-40 (indicated in red ovals), which result in mTORC1 activation. ERK and PDK1 can phosphorylate p90^Rsk1^ (indicated in green ovals), which in turn can phosphorylate and inhibit TSC2 (indicated in red oval). Akt-mediated phosphorylation of GSK-3β also affects the Wnt/β-catenin pathway and EMT. Rapamycin targets mTORC1 and inhibits its activity and also results in inhibition of downstream p70S6K. The effects of rapamycin are complex as long term administration of rapamycin may prevent mTOR from associating with mTORC2 and hence full activation of Akt is prevented. However, rapamycin treatment may result in activation of PI3K, by inhibiting the effects of p70S6K on IRS-1 phosphorylation which results in PI3K and Akt activation. Also rapamycin treatment may result in the activation of ERK in some cells, presumably by inhibition of the p70S6K mediated inhibition of IRS1. These later two effects of rapamycin could have positive effects on cell growth. Energy deprivation will result in the activation of serine/threonine kinase 11 (STK11 a.k.a LKB1) and AMPkinase (AMPK) which can result in TSC2 activation (indicated in red ovals) and subsequent suppression of mTORC1. In contrast Akt can phosphorylate and inhibit the activity of AMPK. Inhibition of PDK-1 activity can also result in activation of mTORC1, presumably by suppression of p70S6K and hence inhibition of IRS1 (indicated in red oval) effects on PI3K activity. The PTEN, TSC1, TSC2 and LKB1 tumor suppressor genes all converge on the mTORC1 complex to regulate protein translation. Thus the Ras/Raf/MEK/ERK and Ras/PI3K/PTEN/Akt/mTOR pathways can finely tune protein translation and cell growth by regulating mTORC1. Rapamycin can have diverse effects on these processes. Also these pathways can interact with the Wnt/β-catenin pathway which is important in developmental processes, EMT and CICs. Upon activation of the Wnt pathway, β-catenin forms a complex with Bcl-9, PYGO, plakoglobulin and TCF/LEF which result in the transcription of critical genes including cyclin D1, c-Myc, SALL4 and PPARδ.

The phosphotidylinositide-dependent kinases (PDKs) are responsible for activation of Akt. PDK1 is the kinase responsible for phosphorylation of T308 [23]. Akt is also phosphorylated by the mammalian target of Rapamycin (mTOR) complex referred to as (Rapamycin-insensitive companion of mTOR/mLST8 complex) mTORC2 [4, 19] (See Figures 2 & 3). Before its discovery, the activity responsible for this phosphorylation event was referred to as PDK2. Therefore, phosphorylation of Akt is somewhat complicated as it is phosphorylated by a complex that lies downstream of activated Akt itself [4, 19]. Thus, as with the Ras/Raf/MEK/ERK pathway, there are feedback loops that serve to regulate the Ras/PI3K/PTEN/Akt/mTOR pathway. Once activated, Akt leaves the cell membrane to phosphorylate intracellular substrates.

After activation, Akt is able to translocate to the nucleus [4, 19, 24] where it affects the activity of a number of transcriptional regulators. CREB [25], E2F [26], nuclear factor kappa from B cells (NF-κB) via inhibitor kappa B protein kinase (Iκ-K) [27], the forkhead transcription factors [28] and murinedouble minute 2 (MDM2) which regulates p53 activity. These are all either direct or indirect substrates of Akt and each can regulate cellular proliferation, survival and epithelial mesenchymal transition (EMT) [4, 11, 19, 28-31]. Aside from transcription factors, Akt is able to target a number of other molecules to affect the survival state of the cell including: the pro-apoptotic molecule Bcl-2-associated death promoter (BAD) [29], and glycogen-synthase kinase-3β (GSK-3β) [30]. GSK-3β regulates β-catenin protein stability. Hence the PI3K/PTEN/Akt/mTOR pathway is connected to the Wnt/β-catenin, p53 and many additional pathways (Figure 3).

Negative regulation of the PI3K pathway is primarily accomplished through the action of the phosphatase and tensin homologue deleted on chromosome ten (PTEN) tumor suppressor proteins. *PTEN* encodes a lipid and protein phosphatase whose primary lipid substrate is PtdIns(3,4,5)P_3_ [31-39]_._ The purported protein substrate(s) of *PTEN* are more varied, including focaladhesion kinase (FAK), the Shc exchange protein and the transcriptional regulators ETS-2 and Sp1 and the platelet-derived growthfactor receptor (PDGFR) [31-33].

PTEN has four primary structural domains. On the amino terminus is the lipid and protein phosphatase domain, which is flanked adjacent to the C2 domain that is responsible for lipid binding and membrane localization. Next are two protein sequences rich in proline (P), glutamic acid (E), serine (S), and threonine (T) (PEST) domains that regulate protein stability. Lastly, PTEN has a PDZ domain, which helps facilitate protein-protein interactions. Mutations within the phosphatase domain have been reported to nullify the endogenous function of PTEN [31, 35]. Thus PTEN is an enticing therapeutic target for activation since it is frequently inactivated in many human cancers through point mutations as well as other means (*e.g*., promoter hypermethylation, gene deletion) and its inactivation results in elevated Akt activity and abnormal growth regulation [31, 35]. Moreover, PTEN can be inactivated by phosphorylation and oxidation in human cancer and which results in elevated Akt activity and abnormal growth regulation [31, 35, 36]. Thus, drugs reactivating PTEN could potentially be very useful in some types of tumors driven by PTEN inactivation.

Another negative regulator of the PI3K pathway is the PH domain leucine-rich repeat protein phosphatase (PHLPP). PHLPP dephosphorylates S473 on Akt which induces apoptosis and inhibits tumor growth [37]. Two other phosphatases, SH2 domain-containing inositol 5'phosphatase (SHIP)-1 and SHIP-2, remove the 5-phosphate from PtdIns(3,4,5)*P*_3_ to produce PtdIns(3,4)*P*_2_ [38-41]. Mutations in these phosphatases, which eliminate their activity, can lead to tumor progression. Consequently, the genes encoding these phosphatases are referred to as anti-oncogenes or tumor suppressor genes.

Next we discuss some of the key downstream targets of Akt that can also contribute to abnormal cellular growth and are key therapeutic targets [4, 19, 35, 42-47]. Akt-mediated regulation of mTOR activity is a complex multi-step phenomenon. Some of these targets and how they interact with the Ras/PI3K/PTEN/Akt/mTOR and Ras/Raf/MEK/ERK pathways are indicated in Figure 3. Akt inhibits tuberous sclerosis 2 (TSC2 or hamartin) function through direct phosphorylation [4, 19, 35, 42]. TSC2 is a GTPase-activating protein (GAP) that functions in association with the putative tuberous sclerosis 1 (TSC1 or tuberin) to inactivate the small G protein Rheb [4, 19, 35, 43, 44]. TSC2 phosphorylation by Akt represses GAP activity of the TSC1/TSC2 complex, allowing Rheb to accumulate in a GTP-bound state. Rheb-GTP then activates, through a mechanism not yet fully elucidated, the protein kinase activity of mTOR when complexes with the Raptor (Regulatory associatedprotein of mTOR) adaptor protein, DEPTOR and mLST8, a member of the Lethal-with-Sec-Thirteen gene family, first identified in yeast [4, 19]. The mTOR/Raptor/mLST8 complex (mTORC1) is sensitive to rapamycin and, importantly, inhibits Akt via a negative feedback loop which involves, at least in part, p70S6K [44]. This is due to the negative effects that p70^S6K^ has on IRS1 [43] (see Figure 3).

The mechanism by which Rheb-GTP activates mTORC1 has not been fully elucidated yet, however it requires Rheb farnesylation and can be blocked by farnesyl transferase (FT) inhibitors.It has been proposed that Rheb-GTP would relieve the inhibitory function of FKBP38 (another component of mTORC1) on mTOR, thus leading to mTORC1 activation [44]. However, more recent investigations did not confirm these findings [45].

Nevertheless, Akt also phosphorylates proline-richAkt-substrate-40(PRAS40), an inhibitor of mTORC1, and by doing so, it prevents the ability of PRAS40 to suppress mTORC1 signalling (recently reviewed in [4, 19, 44]). Thus, this could be yet another mechanism by which Akt activates mTORC1. Moreover, PRAS40 is a substrate of mTORC1 itself, and it has been demonstrated that mTORC1-mediated phosphorylation of PRAS40 facilitates the removal of its inhibition on downstream signaling of mTORC1 [4, 19, 44]. Also Ras/Raf/MEK/ERK signaling positively impinges on mTORC1. Indeed, both p90^Rsk-1^ and ERK 1/2 phosphorylate TSC2, thus suppressing its inhibitory function [4, 19, 44] (See Figure 3). Moreover, recent evidence has highlighted that, in solid tumors, mTORC1 inhibition resulted in ERK 1/2 activation, through p70^S6K^/PI3K/Ras/Raf/MEK [46].

The relationship between Akt and mTOR is further complicated by the existence of the mTOR/Rictor complex (mTORC2), which, in some cell types, displays rapamycin-insensitive activity. mTORC2 has been found to directly phosphorylate Akt on S473 *in vitro* and to facilitate T308 phosphorylation. Thus, mTORC2 can function as the elusive PDK-2 which phosphorylates Akt on S473 in response to growth factor stimulation [47]. Akt and mTOR are linked to each other via positive and negative regulatory circuits, which restrain their simultaneous hyperactivation through a mechanism involving p70^S6K^ and PI3K [4, 19, 35, 44, 48-55]. Assuming that equilibrium exists between these two complexes, when the mTORC1 complex is formed, it could antagonize the formation of the mTORC2 complex and reduce Akt activity [44-46]. Thus, at least in principle, inhibition of the mTORC1 complex could result in Akt hyperactivation. This is one problem associated with therapeutic approaches using rapamycin that block some actions of mTOR but not all.

mTOR is a 289-kDa S/T kinase. It regulates translation in response to nutrients and growth factors by phosphorylating components of the protein synthesis machinery, including p70^S6K^ and eukaryoticinitiation factor (eIF)-4Ebinding protein-1 (4EBP-1), the latter resulting in release of the eukaryotic initiation factor-4E eIF-4E, allowing eIF-4E to participate in the assembly of a translational initiation complex [4, 19, 35, 44]. p70S6K phosphorylates the 40S ribosomalprotein S6, (rpS6), leading to active translation of mRNAs [4, 19, 35, 44]. Integration of a variety of signals (mitogens, growth factors, hormones) by mTOR assures cell cycle entry only if nutrients and energy are sufficient for cell duplication [4, 48-52]. Therefore, mTOR controls multiple steps involved in protein synthesis, but importantly enhances production of key molecules such as c-Myc, cyclin D1, p27^Kip1^, and retinoblastoma protein (pRb) [35].

mTOR also controls the translation of hypoxia-inducible transcription factor-1α (HIF-1α) mRNA [51, 52]. HIF-1α upregulation leads to increased expression of angiogenic factors such as vascular endothelial growth factor (VEGF) and PDGF [4]. Moreover, HIF-1α regulates the glycolytic pathway by controlling the expression of glucose-sensing molecules including glucose transporter (Glut) 1 and Glut3 [51]. By regulating protein synthesis, p70S6K and 4E-BP1 also control cell growth and hypertrophy, which are important processes for neoplastic progression. Hence targeting the mTOR pathway could have many effects on the regulation of cellular growth.

Many of the mRNAs encoding the previously mentioned genes contain 5'untranslated regions which are G+C rich and difficult to translate and referred to as weak mRNAs [35]. 4EP-B1 forms a complex with these mRNAs and other binding factors allowing the translation of these weak mRNAs [35, 53-58]. Rapamycin and mTOR kinase inhibitors suppress the translation of these critical mRNAs involved in cell survival and growth.

### Control of Apoptotic Regulatory Molecules by the Ras/Raf/MEK/ERK and Ras/PI3K/Akt/mTOR Pathways

These two pathways regulate the activity of many proteins involved in apoptosis. In the following section, we will mainly discuss the effects of these pathways elicited by post-translational mechanisms [2, 3, 58-62], although it should be noted that both ERK and Akt also phosphorylate transcription factors that influence the transcription of the Bcl-2 family of genes as well as other important genes involved in the regulation of apoptosis [2, 4, 35, 60-62]. Many of the effects of the Ras/Raf/MEK/ERK and Ras/PI3K/Akt/mTOR pathways on apoptosis are mediated by ERK or Akt phosphorylation of key apoptotic effecter molecules (*e.g*., Bcl-2, Mcl-1, Bad, Bim, CREB, Foxo, Caspase-9 and many others) [2, 4, 35, 59-62]. In addition, both pathways regulate the translation of weak mRNAs. ERK, p90^Rsk-1^, MNK1/2 and p70S6K regulate the phosphorylation of many of the proteins involved in the key complex required for the translation of the weak mRNAs [35,44,56,57]. In some cases, members of the two pathways (*e.g.,* p90^Rsk-1^ and p70^S6K^) will phosphorylate the same molecule in the translation complex at the same site *e.g*. ribosomal protein S6 (rpS6) [57, 58]. However, the kinetics of phosphorylation of rpS6 by the two kinases differs. Thus these two pathways regulate the activity of this translation complex which is responsible for the translation of certain weak mRNAs involved in regulation of apoptosis. Mcl-1 is an example of a weak mRNA and it plays key roles in the regulation of apoptosis.

Aberrant regulation of apoptosis is critically implicated in cancer as well as many other diseases (*e.g*., inflammation, auto-immune diseases). Therefore controlling the activity of the Ras/Raf/MEK/ERK and Ras/PI3K/PTEN/Akt/mTOR pathways have been keen pharmaceutical objectives for many years. The activity of many key components in apoptotic cascades is sensitive to inhibitors that target these pathways.

Akt regulates the apoptotic response to a variety of stimuli via its ability to interact with a number of key players in the apoptotic process [2, 4, 61, 62]. Akt can directly phosphorylate BAD on S136, causing its inactivation preventing it from interacting with anti-apoptotic members of the Bcl-2 family of proteins (Bcl-2, Bcl-X_L_) [29, 62]. Activated Akt can inhibit the release of cytochrome c from the mitochondria, which is a potent activator of the apoptotic caspase cascade [59]. The Akt target, Foxo-3 is capable of upregulating Fas ligand (Fas-L) and Bim, two very important molecules that are potent inducers of apoptosis; however, when inactivated by Akt, Foxo-3 is localized to the cytosol where it is unable to augment expression of these genes [28, 60]. Akt can also phosphorylate Bim which inhibits its proapoptotic activity [61]. In concert, these events caused by Akt activation affect the survival status of the cell.

### Frequent Oncogenic Mutations at Members of these Pathways Result in Activation: Rationale for Therapeutic Targeting of these Pathways

**Figure 4.**
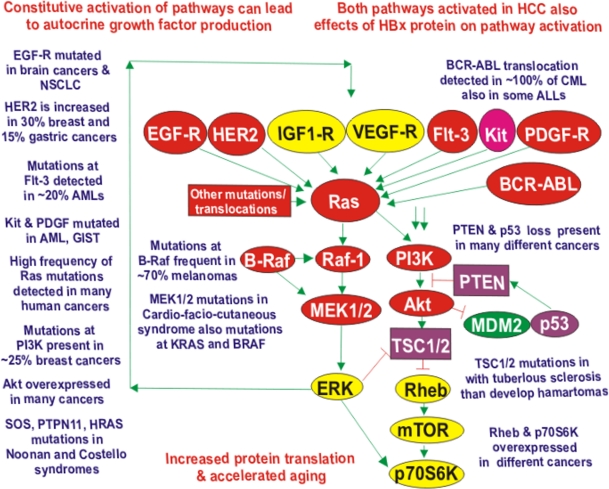
Dysregulated Expression of Upstream Receptors and Kinases Can Result in Activation of the Ras/Raf/MEK/ERK and Ras/PI3K/PTEN/Akt/mTOR Pathways Sometimes dysregulated expression of growth factor receptors occurs by either increased expression or genomic amplifications (*e.g*., *VEGFR*, *EGFR*, *HER2*, *IGF1R*). Mutations have been detected in *EGFR*, *FLT3*, *KIT*, *PDGFR*, *PIK3CA*, *RAS*, *BRAF, MEK1/MEK2, SOS, PTPN11* (indicated in red ovals), and *PTEN* (indicated in a purple square) *.* Akt and Rheb are overexpressed in certain cancers. Other signaling molecules which may be overexpressed (e.g., IGF-1R, VEGF-R, ERK, mTOR, p70S6K) but not necessarily mutated or amplified are indicated in yellow ovals. The MDM2 ubiquitin ligase is indicated in a green oval. The p53 tumor suppressor is one of the most frequently inactivated genes in human cancer and has multiple effects on these pathways and is indicated in a purple oval. Amplifications of *HER2* and *EGFR* are detected in certain cancer types. The *BCRABL* chromosomal translocation is present in virtually all chronic myeloid leukemias (CMLs) and some acute lymphatic leukemias (ALLs). Many of these mutations and chromosomal translocations result in the activation of the Ras/Raf/MEK/ERK and Ras/PI3K/PTEN/Akt/mTOR cascades. These pathways can also be activated by autocrine growth stimulation, the genetic basis of which is frequently unknown. Deregulated expression of these pathways can result in cancer as well as premature aging.

Effective targeting of signal transduction pathways activated by mutations and gene amplification may be an appropriate approach to limit cancer growth, metastasis, drug resistance as well as aging. The Ras/Raf/MEK/ERK and Ras/PI3K/PTEN/Akt/mTOR pathways can be activated by mutations/amplifications of upstream growth factor receptors. The abnormal production of growth factors can result in receptor activation which in turns mobilizes the Ras/Raf/MEK/ERK and Ras/PI3K/PTEN/Akt/mTOR cascades. An illustration of some of the receptors, exchange factors, kinases and phosphatases that are mutated/amplified in human cancer and how they may impact the Ras/Raf/MEK/ERK and Ras/PI3K/PTEN/Akt/mTOR cascades is presented in Figure 4.

Perhaps one of the biggest advances in medical science in the 1980's was the confirmation of the proto-oncogene hypothesis, that predicted that the human genome contains genes related to viral oncogenes which when mutated could cause human cancer [4, 7, 19, 35, 62-84]. Key genetic members of the Ras/Raf/MEK/ERK pathway [*e.g.,RAS*, *RAF*)*, MEK* (rarely) [81-84], the downstream transcription factor (*ETS*) the Ras/PI3K/PTEN/Akt/mTOR (*e.g*.,*PIK3CA*, *AKT, PTEN*) pathway and upstream receptors (*e.g.,ERBB1*(EGF-R)*, ERBB2* (HER2), *PDGFR*, *KIT*, *FLT3, FMS*) were shown to fulfill this hypothesis as they were sometimes mutated/amplified/deleted in specific human cancers. The *RAS*, *RAF*, *PIK3CA, AKT,ERBB1, KIT, FMS* and *ETS* oncogenes are also contained as viral oncogenes in the genomes of certain retroviruses that cause cancer in animals [2, 7, 35, 62]. Furthermore, genetic mutations at these cellular oncogenes often alter sensitivity to specific targeted therapeutic approaches. Thus many of the genes in these two signal transduction pathway can cause cancer under the appropriate conditions.

### Mutation of Upstream Receptors that Activate the Ras/Raf/MEK/ERK and Ras/PI3K/Akt/mTOR Pathways in Human Cancer

Amplification/overexpression of HER2 *[*human epidermal growth factor receptor, *a.k.a*., c-ErbB-2, (*ERBB2*)] is an important cause of sporadic breast cancer that occurs in approximately 30% of breast cancer. HER2 is a receptor tyrosine kinase (RTK) [85]. HER2 can heterodimerize with c-ErbB-3 which has six docking sites for PI3K. While a normal breast cell possesses 20,000 to 50,000 HER2 molecules, amplification of this gene in HER2+ cancers can increase levels of HER2 up to 2,000,000 molecules per cell [85]. Overexpression of HER2 is linked to comedo forms of ductal carcinoma in situ (DCIS) and occurs in approximately 90% of these cases. HER2 overexpression will lead to increased expression of both the Ras/PI3K/Akt/PTEN/mTOR and Ras/Raf/MEK/ERK pathways. Association of genes that regulate signal transduction pathways with breast cancer implies an important role of these pathways in neoplasia.

In acute myeloid leukemia (AML), activation of the Ras/Raf/MEK/ERK and Ras/PI3K/Akt/mTOR pathway can result from mutated upstream targets such as class III RTKs. These include point mutations such as FLT3-internal tandem duplications (FLT3-ITD) and mutated c-KIT, which are present in 35-40% of all AML [2, 4, 35, 62]. Mutations in upstream signaling molecules such as *KIT* and *FLT3* are believed to activate the downstream signal transduction cascades, such as Ras/Raf/MEK/ERK and Ras/PI3K/Akt/mTOR pathways.

### Mutations at *RAS* in Human Cancer

Mutations that lead to expression of constitutively-active Ras proteins have been observed in approximately 20 to 30% of human cancers [7, 63-69]. The frequency of *RAS* mutations and other key genes in the Ras/Raf/MEK/ERK and Ras/PI3K/PTEN/Akt/mTOR pathways in various types of cancers is presented in Table 1. Often point mutations are detected in *RAS* genes in cancer cells from patients which enhance Ras activity. Genome *RAS* amplification or overexpression of Ras, perhaps due to altered methylation of its promoter region, are also detected in some tumors [7, 63-69]. In cholangiocarcinoma, *KRAS* gene mutations have been identified in 45% of examined tumors [7]. Ras mutations are present in up to 20% of AML [67] and are another major cause of activation of this cascade. The frequency of *KRAS* mutations is very high (~80%) in advanced pancreatic cancers [7, 63]. Mutations that result in increased Ras activity often perturb the Raf/MEK/ERK and also the PI3K/PTEN/Akt/mTOR cascades [7].

**Table 1.** Mutations of the Ras/Raf/MEK/ERK and PI3K/PTEN/Akt/mTOR Pathways in Human Cancer

Gene	Cancer Mutated At	Approximate Reported Frequency	Reference
*RAS* genes can be activated by point mutations, gene amplifications and other mechanisms.			
*RAS*	Many different types including pancreatic, acute myeloid leukemia, endometrial, lung, colorectal	20-25% all human cancers, KRAS mutations account for about 85%, NRAS for about 15%, HRAS for <1%.	7
*KRAS*	Pancreatic	90%	7
*HRAS, KRAS, NRAS*	Thyroid (papillary)	60%	7
*HRAS, KRAS, NRAS*	Thyroid (follicular)	55%	7
*KRAS*	Colorectal	45%	7
*KRAS, NRAS*	Seminoma	45%	7
*NRAS, KRAS*	Myelodysplastic syndrome	40%	7
*KRAS*	Non Small Cell Lung Carcinoma	35%	7
*NRAS*	Acute myelogenous leukemia	30%	7
*NRAS*	Liver	30%	7
*KRAS*	Endometrial	20%	7
*NRAS*	Melanoma	15%	7
*HRAS*	Bladder	10%	7
*HRAS*	Kidney	10%	7
			
*BRAF* is activated in approximately 7% all cancers, highest in melanoma, often activated by point mutations.			
*BRAF*	Melanoma	27-70%	72
*BRAF*	Papillary Thyroid	36-53%	72
*BRAF*	Serous Ovarian	30%	72
*BRAF*	Colorectal	5-22%	72
			
*PIK3CA* is often activated by point mutations, also by gene amplification.			
*PIK3CA*	One of the most frequently mutated kinases in human cancer	>30% solid cancers	106, 110
*PIK3CA*	Breast	8-40%	106, 108, 110
*PIK3CA*	Endometrial	23-36%	106, 110
*PIK3CA*	Hepatocellular	36%	106, 108, 110
*PIK3CA*	Colon	19-32%	110
*PIK3CA*	Prostate	29%	31
*PIK3CA*	Glioblastoma	5-27%	106, 110
*PIK3CA*	Head/Neck Squamous Cell	33%	106, 110
*PIK3CA*	Gastric	25%	106, 110
*PIK3CA*	Urinary Track	17%	31
*PIK3CA*	Anaplastic Oligodendroglioma	14%	106, 110
*PIK3CA*	Ovarian	6-12%	110
*PIK3CA*	Intraductal Papillary Mucinous Neoplasm Carcinoma of the pancreas	11%	110
*PIK3CA*	Upper Digestive Track	10%	31
*PIK3CA*	Stomach	8%	31
*PIK3CA*	Esophagus	7%	31
*PIK3CA*	Oral Squamous Cell	7%	110
*PIK3CA*	Pancreas	6%	31
*PIK3CA*	Medulloblastoma	5%	110
*PIK3CA*	Lung	4%	110
*PIK3CA*	Hematopoietic & Lymphoid	4%	31
*PIK3CA*	Skin	3%	31
*PIK3CA*	Anaplastic Astrocytoma	3%	110
*PIK3CA*	Thyroid	2%	31
			
*PTEN* often inactivated by deletion, gene methylation, protein stability and other genetic mechanisms.			
*PTEN*	Endometrial	38%	31
*PTEN*	Central nervous system	20%	31
*PTEN*	Skin	17%	31
*PTEN*	Prostate	14%	31
*PTEN*	Colon	9%	31
*PTEN*	Urinary track	9%	31
*PTEN*	Lung	8%	31
*PTEN*	Ovary	8%	31
*PTEN*	Breast	6%	31
*PTEN*	Hematopoietic & Lymphoid	6%	31
*PTEN*	Thyroid	5%	31
*PTEN*	Stomach	5%	31
*PTEN*	Liver	5%	31
*PTEN*	Upper aerodigestive track	4%	31
*PTEN*	Esophagus	1%	31
*PTEN*	Pancreas	1%	31
			
*AKT is* infrequently mutated in human cancer but *AKT2* gene can undergo amplification in certain cancers.			
*AKT1*	Thryoid	5%	www.sanger.ac.uk/perl.genetics/CGP/cosmic
*AKT1*	Breast	3%	www.sanger.ac.uk/perl.genetics/CGP/cosmic
*AKT1*	Endometrial	3%	www.sanger.ac.uk/perl.genetics/CGP/cosmic
*AKT1*	Ovary	1%	www.sanger.ac.uk/perl.genetics/CGP/cosmic
*AKT1*	Urinary track	1%	www.sanger.ac.uk/perl.genetics/CGP/cosmic
*AKT1*	Prostate	1%	www.sanger.ac.uk/perl.genetics/CGP/cosmic
*AKT1*	Large Intestine	1%	www.sanger.ac.uk/perl.genetics/CGP/cosmic
*AKT1*	Hematopoietic & Lymphoid tissue	1%	www.sanger.ac.uk/perl.genetics/CGP/cosmic
*AKT2*	Head and Neck squamous cell carcinomas	30% amplified	31
*AKT2*	Pancreatic	20% amplified	31
*AKT2*	Ovarian	12% amplified	31
*AKT2*	Breast	3% amplified	31
			
*TSC1/TSC2* is inactivated by point mutations, deletion and other genetic mechanisms. Only *TSC1* is associated with some human cancers.			
*TSC1*	Urothelial Carcinoma	15%	159

A key event in the activation of the Ras protein is farnesylation. Inhibitors that target the enzyme farnesyl transferase (FT) have been developed with the goal of targeting Ras [2]. Clinical testing of FT inhibitors (FTIs) unfortunately has yielded disappointing results. The lack of usefulness of FTIs may be due to multiple reasons. First, there are many proteins that are regulated by FT. Second, although H-Ras is exclusively modified by FT and K-Ras to a lesser extent, N-Ras can also be modified by geranylgeranyltransferase (GGT). This modified N-Ras is still able to support the biological requirement of Ras in the cancer cell. Geranylgeranylation of K-Ras and N-Ras become critical only when farnesylation is inhibited. The majority of *RAS* mutations in humans occur in *KRAS*, which is followed by *NRAS* [7, 67]. The mutation rate at *HRAS* is a distant third [7]. Hence, it is very possible that the effects that FTIs had in initial clinical trials were not due to inhibition of mutant *RAS* genes present in the cell, but in fact resulted from non-specific effects which are related to the first point mentioned. Another important target of FTIs is the Rheb protein (Ras homologue enriched in brain) (See Figure 2). Rheb, another GTP binding/exchange protein, plays key roles in regulating mTORC1 and controlling the efficiency of protein translation [4, 19, 35].

### Mutations at *RAF* in Human Cancer

Prior to 2003, it was believed that the *RAF* oncogenes were not frequently mutated in human cancer. There are three *RAF* genes in humans, (*ARAF, BRAF* and *CRAF* (a.k.a. Raf-1) encoding three distinct proteins with diverse and common functions. With the advent of improved methods of DNA sequencing, it was demonstrated that *BRAF* is frequently mutated in melanoma (27 to 70%), papillary thyroid cancer (36 to 53%), colorectal cancer (5 to 22%), cholangiocarcinoma (22%), ovarian cancer (30%), and a small minority of lung cancer patients (1-3%) [70-75]. *BRAF* mutation occurs in approximately 7% of all cancers [70-73]. In contrast, *CRAF* and *ARAF* are not believed to be frequently mutated in human cancer [80-81].

It was proposed that the structures of B-Raf, Raf-1 and A-Raf kinases may dictate the ability of activating mutations to occur at, and be selected in, the genes encoding these proteins, which can permit the selection of oncogenic forms [75]. These predictions have arisen from the solved structure of B-Raf [75]. Like many enzymes, B-Raf is proposed to have small and large lobes, which are separated by a catalytic cleft. The structural and catalytic domains of B-Raf and the importance of the size and positioning of the small lobe may be critical in its ability to be stabilized by certain activating mutations. In contrast, the functionally similar mutations in *ARAF* and *CRAF* are not predicted to result in small lobe stabilization, this may prevent or hinder the selection of mutations at *ARAF* and *CRAF*, which would result in activated oncogenes [75].

The most frequent mutation detected at the *BRAF* gene is a change at amino acid 600, which converts a Val to Glu (Val600→Glu, V600E) [72]. This *BRAF* mutation accounts for > 90% of the *BRAF* mutations found in melanoma and thyroid cancer. *BRAF* mutations may arise in certain cells that express high levels of B-Raf as a result of hormonal stimulation. Certain hormonal signaling events will elevate intracellular cAMP levels, which result in B-Raf activation, leading to proliferation. Melanocytes and thyrocytes are two such cell types that have elevated B-Raf expression, as they are often stimulated by the appropriate hormones [76]. Moreover, it is thought that B-Raf is the most important kinase in the Ras/Raf/MEK/ERK cascade [75]. In some models, wild-type (WT) and mutant B-Raf are proposed to activate Raf-1, which then activates MEK and ERK [77, 78]. A number of pharmaceutical and biotechnological companies have developed inhibitors that specifically target mutant B-Raf alleles (mutant-allele specific inhibitors), which do not inhibit WT B-Raf [3].

In many cancers with *BRAF* mutations, the mutations are believed to be initiating events and also the driver mutations, but are not sufficient for complete neoplastic transformation [35, 65, 66, 72-75]. Mutations at other genes (*e.g*., in components of the Ras/PI3K/PTEN/Akt/mTOR pathway) have been hypothesized to be also necessary for malignant transformation in some cancers. Moreover, there may be certain situations where certain potent *BRAF* mutations (Val^600^→Glu) and *RAS* mutations are not permitted in the same cell, as they might result in hyperactivation of Ras/Raf/MEK/ERK signaling and expression, which could lead to cell cycle arrest [75]. In contrast, there are other cases that require both *BRAF* and *RAS* mutations for transformation. The *BRAF* mutations in these cases may result in weaker levels of B-Raf activity which is insufficient for abnormal proliferation [65, 66, 75, 77, 78]. It should be pointed out that *RAS* mutations may also result in activation of the Ras/PI3K/Akt/mTOR pathway.

Different *BRAF* mutations have been mapped to various regions of the B-Raf protein. Mutations at *BRAF* that result in low kinase activity may signal through Raf-1 [75, 77, 78]. Heterodimerization between B-Raf and Raf-1 proteins may allow the impaired B-Raf to activate Raf-1. Other mutations, such as Asp^593^→Val, may activate alternative signal transduction pathways [75].

One study has observed that mutated alleles of *CRAF* are present in therapy-induced acute myelogenous leukemia (t-AML) [80]. This t-AML arose after chemotherapeutic drug treatment of breast cancer patients. The mutated *CRAF* genes were transmitted in the germ line, thus, they were not spontaneous mutations in the leukemia, but they may be associated with the susceptibility to induction of t-AML in the breast cancer patients studied. Subsequent studies demonstrated that blast cells from patients with the *CRAF* germline mutations also had loss of the tumor and metastasis suppressor Raf kinase inhibitor protein (RKIP) [81]. The importance of RKIP was determined by transfection experiments with either siRNA directed against RKIP or expression vectors overexpressing RKIP [81]. The levels of RKIP were determined to influence the levels of *CRAF*-mediated transformation as high levels of RKIP suppressed *CRAF*-mediated transformation, while low levels enhanced *CRAF*-mediated transformation [81]. Decreased RKIP expression has also been observed in some cutaneous squamous cell carcinomas which also displayed decreased BRAF expression [79]. Thus mutation at both *BRAF* and *CRAF* have been detected in certain cancer patients and other studies have shown that the levels of mutant and WT B-Raf, Raf-1 and RKIP will influence the levels of transformation observed, hence there is a strong basis for the development of Raf inhibitors [3].

Mutations downstream of Raf in the Ras/Raf/MEK/ERK cascade have not been frequently detected in human cancer although there are some rare germline mutations detected at *MEK1* and *MEK2* in cardiofaciocutaneous syndrome (CFC) [82]. There are also mutations at other components of the Ras/Raf/MEK/ERK pathway including *KRAS* and *BRAF* in CFC. There are mutations at components of the Ras/Raf/MEK/ERK pathway in the related Costello and Noonan syndromes, including: *SOS*, and *PTPN11* (Shp2) in Noonan syndrome and *HRAS* mutations in Costello syndrome [83]. These germline mutations confer sensitivity to MEK inhibitors. *MEK1* but not *ERK2* mutations have been observed in some melanomas and colon carcinomas [84].

### Activation of the Ras/Raf/MEK/ERK Cascade in the Absence of Mutations in the Pathway

Hepatocellular carcinoma (HCC) is the fifth most common cancer worldwide and the third most prevalent cause of cancer mortality, accounting for approximately 6% of all human cancers and more than 600,000 deaths annually worldwide [85, 86]. Although the clinical diagnosis and management of early-stage HCC has improved significantly, HCC prognosis is still extremely poor. Therefore, investigating HCC pathogenesis and finding new diagnostic and treatment strategies is important.

Signaling via the Ras/Raf/MEK/ERK cascade plays a critical role in liver carcinogenesis [86-91]. Although mutations of Ras and Raf occur infrequently in HCC, a recent study demonstrated that activation of Ras pathway occurred in 100% of HCC specimens analyzed when compared with non-neoplastic surrounding tissues and normal livers [91].

In addition, activation of Ras/Raf/MEK/ERK pathway in HCC may be due to down-regulation of Ras inhibitors Sprouty and the Sprouty-related protein with Ena/vasodilator-stimulated phosphoprotein homology-1 domain (Spred-1) and Spred-2 proteins [92, 93]. It has been shown that the expression of Spred-1 and -2 in human HCC tissues is frequently decreased, in comparison to adjacent non-tumorous tissues. This decreased expression inversely correlated with the incidences of tumor invasion and metastasis [92]. Moreover, ectopic Spred expression inhibited HCC cell proliferation both *in vitro* and *in vivo*, which was associated with reduced ERK activation, suggesting that Spred could be both a novel prognostic factor and a new therapeutic target for human HCC [93].

Down-regulation of RKIP expression is a major factor in activation of the Ras/Raf/MEK/ERK pathway during human hepatocarcinogenesis [94]. These studies indicate the complex interplay of various genes that serve to regulate the Ras/Raf/MEK/ERK pathway. Deregulation of their expression by various mechanisms (*e.g*., promoter methylation, point mutations, post-translational mechanisms) may result in Ras/Raf/MEK/ERK pathway activation in the absence of detectable mutations at either *Raf* or *MEK*. Hence, the Ras/Raf/MEK/ERK cascade is a therapeutic target in HCC [3, 95, 96].

Obesity is another important contributing factor for the development of HCC [97]. The important role of Ras/Raf/MEK/ERK signaling has also been suggested for HCC progression in obese patients. A possible explanation for risk associated between obesity and HCC comes from the study of Saxena *et al*., which for the first time demonstrated that leptin, akey molecule involved in the regulation of energy balance and body weight control, promotes HCC growth and invasiveness through activation of Ras/Raf/MEK/ERK signaling [98].

Other well known risk factors for HCC such as hepatitis B and Cviruses (HBV and HCV) also utilize the Ras/Raf/MEK/ERK pathway for the control of hepatocyte survival and viral replication [99]. Among the four proteins encoded by HBV genome, HBx is involved in heptocarcinogenesis. HBx activates Ras/Raf/MEK/ERK signaling cascade [100]. Among HCV components, the core protein has been reported to activate the Ras/Raf/MEK/ERK pathway and thereby might contribute to HCC carcinogenesis [101, 102]. Therefore, these studies suggest that the Ras/Raf/MEK/ERK pathway is a novel therapeutic target that could be exploited for the treatment of HCC resulting from HBV and HCV infection. microRNAs (miRNAs) may play a key role in regulating HCV translation [103]. Protein translation is regulated by the Ras/Raf/MEK/ERK and Ras/PI3K/PTEN/Akt/mTOR pathways and may be a therapeutic target for HCC [104]. The interacting Wnt/β-catenin pathway also has effects on HCC [105].

### Mutations at *PIK3CA* in Human Cancer

The PI3K p110α catalytic subunit (*PIK3CA*) gene is currently the most frequently mutated kinase in human cancer. *PIK3CA* is mutated in approximately 25% of breast, 32% of colorectal, 30% of endometrial, 27% of brain, 25% of gastric, 4% of lung cancers [106-110] (Table 1). These mutations are clustered in small hot-spot regions within the helical (E542, E545) and kinase (H1047) domains [106-110]. The locations of these mutations have been recently critically evaluated [110]. These mutations frequently result in activation of its kinase activity [110]. Furthermore increased expression of the Ras/PI3K/Akt/mTOR pathway also occurs frequently in some cancers as the *PIKC3A* gene is amplified in approximately 40% of ovarian cancers [109].

Activation of PI3K/PTEN/Akt/mTOR signaling through mutation, inactivation or silencing of pathway components occurs in various malignancies, including liver cancer [111]. Deregulation of this pathway has clinical importance in HCC. For example, recent data from genomic sequence of HCC samples identified mutations in *PIK3CA* in 50% of patients with poor prognosis, survival length < 3 years following partial liver resection, and only 10% of the HCC patients with a good prognosis had mutation in *PIK3CA* [111]. The identified mutations were restricted to residues H1047 in 61.1%, to E545 in 33.3%, and to E542 in 5.5% of cases, and as a consequence this result in gain of enzymatic function and consequently in oncogenic activity of PI3K [111].

### Mutations at *PTEN* in Human Cancer

Germline PTEN mutations are present in approximately 80% of patients with Cowden syndrome [112]. This disease, which is also known as multiple hamartoma syndrome, is another familial syndrome that includes many different types of cancer conditions including early onset breast cancer. Mutations have been reported to occur at *PTEN* in breast cancer in varying frequencies (5-21%) [113, 114]. Loss of heterozygosity (LOH) is probably more common (30%) [114]. Mutations at certain residues of PTEN, that are associated with Cowden's disease, affect the ubiquitination of PTEN and prevent nuclear translocation. These mutations leave the phosphatase activity intact [115]. Inhibition of PTEN activity leads to centromere breakage and chromosome instability [34]. Thus PTEN has diverse activities.

Akt and mTOR phosphorylation are frequently detected in ovarian and endometrial cancers. An early occurrence in endometrial cancer is the loss of functional PTEN activity by mutation or other mechanisms, this occurs in approximately 40-80% of patients [116]. Since the loss of PTEN results in activation of Akt, that in turn up-regulates mTOR activity, cancer cells deficient in PTEN are thought to be major targets of mTOR inhibitors.

The best evidence that strongly supports the connection between PTEN-suppression and liver carcinogenesis comes from genetic studies. All mice with *PTEN*-deficient hepatocytes exhibited liver adenomas and 66% of them developed HCC [117]. In these mice, hepatocytes were hyperproliferative and displayed an abnormal activation of Akt [117]. Furthermore, although mutations in the *PTEN* gene rarely occur in HCC, frequent loss of heterozygosity of *PTEN* allele has been identified in 20-30% of HCC patients [118-121]. In addition, down-regulation of PTEN expression may be partly due to *PTEN* promoter methylation [122]. PTEN expression plays a critical role in HCC progression and patient's outcome. Patients with high expression of PTEN had a significantly better overall survival than patients with low PTEN expression [123]. As mentioned above, hepatitis viruses protect hepatocytes from apoptotic cell death by promoting the activation of Ras/PI3K/Akt/mTOR survival pathway [124, 125]. Among the four proteins encoded by HBV genome, HBx has been reported to be involved in hepatocarcinogenesis. It has been reported that HBx expression downregulated PTEN expression in hepatocytes [125]. In contrast, PTEN expression in liver cells downregulated HBx-induced PI3K and Akt activities [126]. Therefore, these studies suggest the possible use of PTEN as a target in therapeutic approaches for the treatment of at least those HCC caused by HBV infection.

In some cancer settings, *PTEN* and *BRAF* mutations appear to interact. Two recent papers have highlighted the hypothesis of mutant *BRAF*- and *PTEN*-loss-driven carcinogenesis in mouse models. In a study by Dhomen *et al.*, inducible expression of B-Raf^V600E^ was sufficient to induce multiple melanocytic lesions including skin hyperpigmentation, dysplastic nevi and melanoma [127]. Tumor cells from these B-Raf^V600E^ mice displayed both melanoma growth and melanocyte senescence in this system. Approximately 70% of these mice developed melanomas that exhibited histological and molecular characteristics similar to that of human melanoma and were able to colonize the lungs in nude mice [127]. In contrast, another group of researchers generated mice that conditionally-expressed melanocyte-specific B-Raf^V600E^ that were only able to induce benign melanocytic hyperplasias and were unable to progress any further over a 15-20 month period [128]. However, B-Raf^V600E^ expression in a *PTEN* gene-silenced background led to the production of melanoma with 100% establishment, short latency and metastasis to lymph nodes and lungs. This development was prevented by the treatment of mice with either the mTOR inhibitor rapamycin or the MEK1/2 inhibitor (PD0325901). Moreover, while combination treatment with rapamycin or PD0325901 led to the reduction of established tumors, upon termination of drug treatment the melanomas reappeared the presence of drug resistant melanoma-initiating cells in these mice. Overall, these two papers further validated the mutant B-Raf/MEK/ERK and the PI3K/Akt/mTOR pathways, as promising therapeutic targets in melanoma.

Mutations and hemizygous deletions of *PTEN* have been detected in AML and non Hodgkin's lymphoma (NHL) and other cancers [129, 130]. Thus the PTEN gene is a critical tumor suppression gene, frequently mutated in human cancer.

### Alterations of PTEN Expression in Human Cancer

Phosphorylation (inactivation) of PTEN has been associated with increased Akt-activity. Although many groups have investigated the PTEN-phosphorylation status in leukemia and lymphoma, its relevance concerning Akt-activation is still not clear [129-133]. PTEN phosphorylation as well as low or absent PTEN expression has been observed in AML.

Furthermore, the level of PTEN expression does not always correlate with the degree of phosphorylation of Akt [129]. Although the picture concerning PTEN-inactivation and corresponding Akt-activation is not clear, *in vivo* studies indicate, that PTEN dysregulation promotes leukemogenesis. *PTEN*-deficient hematopoietic stem cells display dysregulated cell cycle progression, and the mice develop a myeloproliferative disease which leads to leukemic transformation [131]. In T-acute lymphoblastic leukemia (T-ALL), PTEN-down-regulation is also closely correlated with Akt-activation [132, 133]. To discern the role of PTEN for Akt-activation, it may be useful to exclude concomitant causes for Akt-activation such as mutant upstream targets and to include the investigation of regulators of PTEN such as c-Myc and Notch/Hes1 [132, 133].

PTEN promoter methylation leads to low PTEN expression [134]. In one study, 26% of primary breast cancers had low PTEN levels that correlated with lymph node metastases and poor prognoses [135].

Other mechanisms important in the regulation of PTEN are miRNAs. Certain miRNAs have been shown to regulate PTEN protein expression. mi-214 induces cell survival and may contribute to oncogenesis and drug resistance (see below) by binding the 3'untranslated region (3'UTR) of PTEN which prevents PTEN mRNA translation and leads of overexpression of downstream Akt [136].

### Mutations at *SHIP* Phosphatase in Human Cancer

The SHIP-1 phosphatase has been implicated as a suppressor of hematopoietic transformation as it basically can prevent Akt-activation [137]. SHIP-1-deficient mice develop a myeloproliferative disease [138] and an inactivating point mutation (*SHIP V684E*) has been observed in approximately one of thirty AML cases [137]. Also another mutation, *SHIP Q1154L*, has been observed in AML, but was even less frequent (1 of 192 cases) [138]. Though some studies confirmed, that SHIP-1 is a leukemia suppressor [137, 138] it is unlikely that *SHIP1* mutations are a frequent cause of Akt-activation in AML. Disruption of *PTEN* or *SHIP* activity by various genetic mechanisms could have vast effects on different processes affecting the sensitivity of different cancers to various therapeutic approaches.

### Mutations of *AKT* in Human Cancer

The roles that Akt plays in cancer are complex. Akt can be activated by genetic mutations, genome amplifications and more commonly by mutations in upstream signaling components. Amplification of Akt-2 was observed in human ovarian carcinomas [139]. Increased levels of Akt are detected in carcinomas of the breast, ovary and prostate and are associated with a poorer prognosis in comparison with tumors that do not display increased levels of expression. Akt is a member of a multigene family that consists of *AKT1*, *AKT2* and *AKT3*. *AKT1* has been reported to be mutated in some breast, colorectal, melanoma and ovarian cancers [140-142] (see below). *AKT2* is not mutated frequently in human cancer. *AKT2* is amplified in certain cancers (*e.g*., 12.1% ovarian and 2.8% breast carcinomas) [142]. A recent report documents the mutation of *AKT3* in some melanoma samples [143].

*AKT1* is mutated in 2 to 8% of breast, 6% of colorectal and 2% of ovarian cancers samples examined in one study [140]. This study documented an Akt mutation that results in a glutamic acid (E) for a lysine (K) substitution at amino acid 17 (E17K) in the PH domain. Cells with this *AKT1* mutation have not been observed to have mutations at *PIK3CA*; a similar scenario is also frequently observed with *RAS* and *BRAF* mutations [144]. This *AKT1* mutation alters the electrostatic interactions of Akt-1 which allows it to form new hydrogen bonds with the natural PtdIns ligand [140]. The PH domain mutation confers many different properties to the *AKT1* gene. Namely the mutant *AKT1* gene has: 1) an altered PH domain conformation, 2) is constitutively-active, 3) has an altered cellular distribution as it is constitutively-associated with the cell membrane, 4) morphologically transforms Rat-1 tissue culture cells and 5) interacts with c-Myc to induce leukemia in Eμ-Myc mice (Eμ = Enhancer of immunoglobulin μ gene, Myc = Myc oncogene originally isolated in avian myelocytomatosis virus) [140]. This PH domain mutated *AKT1* gene does not alter its sensitivity to ATP competitive inhibitors, but does alter its sensitivity to allosteric kinase inhibitors [140]. These results demonstrate t hat targeting the kinase domain of Akt may not be sufficient to suppress the activity of various *AKT* genes that have mutations in the PH domain.

### Alterations of Akt Expression in Human Cancer

Akt is often upregulated in cancer cells and its overexpression is associated with a poor prognosis. Increased expression of Akt can result from activating *PIK3CA* mutations or elimination or decrease in PTEN activity. Elevated Akt expression has also been associated with the pathology of pancreatic, glioma and prostate cancers [145-148].

Pancreatic cancer cells have elevated IGF-1R expression and it is well known that Akt regulates IGF-1R expression [149]. This Akt effect on IGF-1R has been suggested to be responsible for the invasiveness of pancreatic cancer cells. Active Src can also activate Akt, and both Src and Akt up-regulate IGF-1R expression in this cancer. It has been demonstrated that IGF-I is expressed in the surrounding stromal cells but not in the cancer cells. This IGF-1 expression may serve as a paracrine growth factor to activate the IGF-1R pathway and the downstream Ras/PI3K/Akt/mTOR pathway in pancreatic cells.

Cyclooxygenase-2 (COX-2) is expressed at high levels in some primary endometrial tumors and is associated with an aggressive phenotype [150]. Akt is elevated and PTEN is often mutated in these cancers. Recently, NF-κB activation has been shown to have oncogenic effects important in the control of apoptosis, cell cycle, differentiation and cell migration. Akt may exert its effects through the NF-κB pathway and COX-2 is the regulator of this pathway. Akt regulates *COX2* gene and protein expression in endometrial cancers. This study was undertaken to examine the involvement of Akt in the regulation of NF-κB and COX-2 [150]. The expression of both inhibitor of NF-κB (IκB) and phosphorylated IκB were increased in the cells containing mutant *PTEN* genes. In contrast, there was no difference in NF-κB protein abundance between the cell lines, which differed in PTEN gene status. IκB phosphorylation by the PI3K pathway was inhibited by the PI3K inhibitors Wortmannin and LY294002. There was less NF-κB nuclear activity, less COX-2 expression and more apoptosis after inhibition of the PI3K pathway. Dominant negative (DN) Akt blocked IκB phosphorylation and decreased COX-2 expression. In contrast, introduction of constitutively-active Akt induced IκB phosphorylation and up-regulated COX-2 expression.

When *PTEN* is mutated, Akt signals via the NF-κB/IκB pathway to induce COX-2 expression in endometrial cancer cells. COX-2 can inhibit apoptosis, increase angiogenesis, and promote invasiveness. COX-2 also promotes inflammation/immunosuppression and conversion of procarcinogens into carcinogens that contribute to tumorigenesis and a malignant phenotype. This study demonstrated that Akt signals via the NF-κB/IκB pathway to induce *COX2* gene and protein expression in endometrial cancer [150].

Elevated Akt activity can also result in increased phosphorylation of mTOR. mTOR was found to be phosphorylated in AML blasts, along with its two downstream substrates, p70^S6K^ and 4EBP-1, in a PI3K/Akt-dependent fashion [151]. Nevertheless, others failed to detect any relationship between PI3K/Akt signalling upregulation and p70^S6K^ phosphorylation in AML primary cells [152]. This might occur via the Ras/Raf/MEK/ERK pathway activating mTOR via ERK phosphorylation [152]. The Ras/Raf/MEK/ERK pathway is frequently activated in AML [153]. Thus treatment of AMLs with Raf and MEK inhibitors is being activated investigated [3, 154, 155].

Akt is activated in HCC, which results in enhanced resistance to apoptosis through multiple mechanisms [101, 156-158]. As an example, activation of the Akt pathway suppresses transforming growth factor-β (TGF-β) induced apoptosis and growth-inhibitory activity of CCAAT/enhancer binding protein alpha (CEBP-α). Activation of Akt is a risk factor for early disease recurrence and poor prognosis in patients with HCC [156]. Several mechanisms may be responsible for the activation of Akt. The high frequency of *PIK3CA* mutations and/or its upregulation in patients with shorter survival might be responsible for the Akt hyperactivation found in HCC with poor prognosis [118-124]. Selective epigenetic silencing of multiple inhibitors of the Ras pathway seems also to be responsible for the activation of Akt found in HCC [111]. Moreover, impaired expression of PTEN is involved in the regulation of Akt activity. Activation of Akt signaling and reduced expression of PTEN has been reported in 40%-60% of human HCC cases [111, 118-124]. Some well known risk factors, HBV and HCV seem to utilize the Ras/PI3K/PTEN/Akt/mTOR pathway for the control of hepatocytes survival and viral replication [157, 158]. Taken together, these data suggest that Ras/PI3K/Akt/mTOR pathway may represent an important therapeutic target for the treatment of HCC among patients with differing etiologies that lead to the development of this aggressive tumor.

### Mutations of *TSC1/TSC2* Genes in Human Cancer

Mutations in the tumor suppressor genes *TSC1* and *TSC2* are associated with a dominant genetic disorder, tuberous sclerosis [42, 159]. Patients with mutant *TSC* genes develop benign tumors (hamartomas). In contrast to Cowden's patients who have germline mutations at *PTEN* and the patients have a high propensity to develop multiple malignancies, TSC patients rarely develop multiple malignant cancers, and if they do develop malignant cancers they are usually either renal cell carcinomas (RCCs) or angiomyolipomas [159]. This has been hypothesized to result from a lack of activation of Akt in cells that have mutant *TSC1* or *TSC2* as mTOR activity is expressed at higher levels which results in inhibition of Akt, perhaps via the effects of p70S6K oninsulin regulated substrate-1 (IRS1) (Figure 3). *TSC1* has been shown to be mutated in approximately 15% of urethelial carcinomas (bladder cancers) [159].

### Altered Expression of Components Downstream of mTOR in Human Cancer

mTOR regulates translation by phosphorylating components of the protein synthesis machinery, including p70S6K and 4E-BP1 (eukaryotic initiation factor 4E-binding protein 1) [160, 161]. p70S6K phosphorylates the 40S ribosomal protein, rpS6, leading to active translation of mRNAs [4]. In contrast, 4E-BP1 phosphorylation by mTORC1 on several amino acidic residues (S37; T46; S65; T70) results in the release of the eukaryotic initiation factor 4E (eIF4E) [5]. mRNAs differ in their ability to be translated; the length and sequence of the 5' UTR largely dictates the efficiency with which an mRNA transcript will be translated. Most mRNAs contain short, unstructured GC-poor 5' UTRs and are efficiently translated. In contrast, long, GC-rich sequences in the 5' UTR often hinder the ability of the eIF-4E complex to efficiently scan and initiate translation at the start codon [4, 19, 35]. These are called weak mRNAs as previously discussed. Consequently, under normal circumstances these mRNAs are not efficiently translated, and are considered “weak” mRNAs [4, 19, 35]. However, upon Akt-mediated activation of mTOR, these latter mRNAs are highly and disproportionately translated. Interestingly, many of these weak mRNAs molecules encode oncogenic proteins involved in cell proliferation or survival (*e.g.,* c-Myc, Mcl-1, cyclin-D, VEGF and survivin). These oncogenic mRNAs are therefore tightly regulated at the translation level and their accumulation in cancer cells strongly contributes to the malignant phenotype.

Several key proteins that are overexpressed as a consequence of mTOR activation include: c-Myc[162-164], cyclin D1 [164], and VEGF [165] and others. Cyclin D1 has been reported to be overexpressed in prostate cancer xenografts and metastases [166], while early stage prostatic lesions possess much lower levels of the protein [167]. A number of reports support the notion that mTOR signaling is a prominent feature of cancer progression and aging, as recurrent tumors have altered expression of a number of molecular targets of rapamycin including the above mentioned genes which encode “weak” mRNAs [168-171]. Hence mTOR inhibitors such as rapamycin may be effective in cancer therapy.

One central molecule involved in cell growth is p70S6K which is regulated by both the Ras/PI3K/PTEN/Akt/mTOR and Ras/Raf/MEK/ERK pathways [4]. The p70S6K gene is amplified in approximately 9% of primary breast cancers and elevated levels of its mRNA transcripts are found in about 41% of the tumors [173, 174]. It is known that some PTEN-deficient cells and tumors that are purported to grow in response to activated Akt are hypersensitive to mTOR inhibitors. p70S6K activity is reduced by mTOR inhibitors in PTEN-deficient cells and transgenic PTEN^+/−^ mice [175, 176].

### Involvement of the Ras/Raf/MEK/ERK and Ras/PI3K/PTEN/Akt/mTOR Pathways in Hormone-Independent Prostate Cancer

The progression of prostate cancer from androgen-dependent to androgen-independent tumors involves the alteration of the androgen receptor and/or the activation of pro-survival pathways, namely those of the Ras/Raf/MEK/ERK and Ras/PI3K/PTEN/Akt/mTOR signaling cascades [177, 178]. Research has shown that inhibition of one or both of these pathways has a more profound effect on tumor cell development and death making them very attractive as combinational targets in prostate cancer therapy. In the study by Wu *et al*., cells from the androgen-dependent cell line LNCaP were able to differentiate into neuroendocrine type cells upon androgen withdrawal from the culture media [177]. This differentiation was marked by a change in cellular morphology and expression of the chromogranin and neuron-specific enolase (NSE), as well as an increase in phosphorylated ERK and Akt. Inhibition of the Ras/PI3K/PTEN/Akt/mTOR pathway with the PI3K inhibitor LY294002 and the mTOR inhibitor Rapamycin reduced the expression of these neuroendocrine specific cell markers however the use of the MEK inhibitor U0126 appeared to have no effect [177]. In another study, *Nkx3.1;Pten* mutant mice were used as a preclinical model for the effects that inhibition of both Ras/Raf/MEK/ERK and Ras/PI3K/PTEN/Akt/mTOR pathways would have on hormone-dependent and -independent prostate cancer growth [178]. The *Nkx3.1;Pten* mutant mouse model resembles that of human prostate cancer progression in which spontaneous PIN lesions form and progress to adenocarcinomas and eventually hormone refractory tumors upon androgen deprivation. Treatment of tumors from these mice both *in vivo* and *in vitro* with rapamycin and the MEK inhibitor PD0325901 were able to synergistically decrease their respective target pathway's activation more effectively and at a lower IC_50_ compared to treatment with each agent alone [179]. Interestingly, although combination inhibitor therapy was somewhat effective at reducing tumor size and proliferation in the androgen-intact mouse model, the highest reduction in tumor growth from therapy was observed in the androgen-deficient mice [178]. In addition to the mouse study these authors were able to show, using human patient tissue microarrays, that aberrant activation of some of the Ras/PI3K/PTEN/Akt/mTOR pathway components (Akt, mTOR, p70S6K) are frequent in progressed human prostate tumors. In addition, activation of the Ras/Raf/MEK/ERK pathway coincides with a high percentage of these tumors as well, suggesting that combination inhibitor treatment along with hormone ablation could prove useful in human prostate cancer therapies [178].

### Interactions of p53 and the Ras/Raf/MEK/ERK and PI3K/PTEN/Akt/mTOR pathways

Ras/Raf/MEK/ERK and Ras/PI3K/PTEN/Akt/mTOR pathways are often regulated by the tumor suppressor p53. Furthermore p53 activity is likewise regulated by the Ras/Raf/MEK/ERK and Ras/PI3K/PTEN/Akt/mTOR pathways. p53 is a critical tumor suppressor gene which encodes a transcription factor that is frequently mutated in human cancer [179-195]. P53 regulates the transcription of many genes whose protein products play critical roles in cell cycle progression, apoptosis, senescence, quiescence and aging. p53 is often activated after chemotherapeutic drug treatment and DNA damage [182, 183, 193-195] There are complex interactions between p53, DNA damage responses and these two signaling pathways [180-195]. Akt can phosphorylate MDM-2 which leads to its proteasomal degradation and prevents it ability to interact with and destabilize p53 [4]. The p53 and MDM families of genes are critically involved in the response to DNA damage [183-185], apoptosis [185], senescence [186], metastasis [188], autophagy [190], chemosensitivity [191, 195] and cellular aging [179, 181, 182]. Thus the ability to fine tune these pathways could significantly advance human health. MDM-2 inhibitors such as Nutlin-3A increase p53 stability [179]. p53 can affect the transcription of the PTEN and other important gene involved in cell cycle regulation (*e.g*., p21^Cip-1^), apoptosis (*e.g*., Bax, Noxa, Puma) and cellular senescence [e.g., Yippee-like-3 (YPEL3)], [180, 184-186, 192]. Thus reactivation of p53 expression could enhance PTEN gene expression and hinder activation of Akt.

The Ras/Raf/MEK/ERK pathway can regulate p53 activity and p53 can also induce the activity of key components of this pathway [196-198]. ERK can phosphorylate p53 and alter its activity. Moreover, chemotherapeutic drugs such as doxorubicin can induce the p53 activity that in turn can activate the expression of the discoidindomain receptor (DDR) which can induce Ras and the downstream Ras/Raf/MEK/ERK and Ras/PI3K/PTEN/Akt/mTOR pathways [196-198].

In certain scenarios, increased p53 expression after chemotherapeutic drug treatment may lead to increased Ras/Raf/MEK/ERK and Ras/PI3K/PTEN/Akt/mTOR pathways activation, resulting in an undesired pro-proliferative effect [195]. This may occur in certain cancer initiating cells (CICs) and be a component of their inherent drug resistance. In addition, Akt has critical roles in regulation of cell cycle progression [199-202] Thus in those therapeutic scenarios where increased p53 activity is desired, it may also be prudent to also consider treatment with either a Raf or MEK inhibitor to decrease the activation of this pro-proliferative pathway.

### Novel Roles of the Ras/Raf/MEK/ERK and Ras/PI3K/PTEN/Akt/mTOR Pathways in Cancer and Aging

In the previous sections, we have discussed the mechanisms of activation of the Ras/Raf/MEK/ERK and Ras/PI3K/PTEN/Akt/mTOR pathways in human cancers, predominantly by mutational based mechanisms. Recently the Ras/Raf/MEK/ERK and Ras/PI3K/PTEN/Akt/mTOR pathways have been shown to have roles in cancer stem cells, senescence, aging and sensitivity to targeted therapy [203-245]. These additional functions of these pathways expand their important in human health.

An area of intense interest in cancer biology is the cancer stem cell, more appropriately referred to as the cancer initiating cell (CIC) [203-211]. The concept that the Ras/Raf/MEK/ERK and Ras/PI3K/PTEN/Akt/mTOR pathways serve as key pathways in regulating CIC survival is beginning to emerge. CICs have unique properties as they can be both quiescent and also resistant to chemotherapeutic and hormonal based drugs [203]. However, under certain conditions, they resume proliferation and hence should be potentially susceptible to Ras, Raf, MEK, PI3K, Akt or mTOR inhibitors.

The *PTEN* gene has been shown to exert effects on CICs, especially in hematopoietic and breast cells [204-209]. In conditional *PTEN* knock-out mice, upon inactivation of *PTEN*, there is a transient increase in hematopoietic CICs and a myeloproliferative disease develops and the mice subsequently develop leukemia after 4-6 weeks [204]. If the mice are treated with rapamycin, the myeloproliferative disorder and leukemia are prevented. The initial leukemic CICs that arise after conditional *PTEN* deletion by themselves are not able to induce leukemia upon transfer into severe combined immunodeficiency (SCID)-recipient mice, but if the leukemic CICs were derived from the *PTEN*-conditional mice that had developed leukemia, they were able to transfer leukemia to the SCID-recipient mice, which could be prevented by rapamycin treatment [204]. Also the normal hematopoietic stem cells from the *PTEN-* conditional knock-out mice could repopulate the hematopoietic cell component of irradiated mice treated with rapamycin indicating that it is possible to selectively eliminate leukemic CICs.

PTEN also plays important roles in breast CICs [205, 206]. If *PTEN* is mutated, Akt phosphorylates and inactivates glycogen synthetase kinase 3 β (GSK-3β) which in turn regulates the activity of the Wnt/β-catenin pathway (Figure 3), as β-catenin is not phosphorylated by GSK-3β and not degraded. β-catenin can localize to the nucleus, perhaps due to Akt-mediated phosphorylation at S552 and exert its effects. β-catenin can then promote the expression of many genes such as cyclin D, c-Myc, SALL4 and peroxisome proliferator-activatedreceptor-δ (PPARδ) which are important in cell survival and EMT. The Ras/PI3K/PTEN/Akt/mTOR pathway performs key roles in the regulation of the size of the Aldefluor-positive cell population that are enriched in breast CICs. Treatment with the Akt inhibitor perifosine was able to target these cells both in *in vitro* and xenograft models [206]. In contrast, the chemotherapeutic drug docetaxel was unable to target the Aldefluor-positive cells and these cells were not sensitive to mTOR inhibitors, suggesting that the mTOR pathway was not involved in these breasts CIC. The studies by Korkaya *et al.* [206] indicate that targeting some breast CICs with perifosine may eliminate these cells that are responsible for tumor reappearance. Other studies have shown that breast CICs are resistant to chemotherapeutic drugs [212-214].

We have observed that some drug resistant breast cells that express properties similar to CICs display elevated activation of the Ras/Raf/MEK/ERK and Ras/PI3K/PTEN/Akt/mTOR signaling cascades and that CICs can be isolated from these cell populations [215-217]. Our recent data suggests these CICs are more sensitive to MEK and mTOR inhibitors than either the parental or drug resistant cells from which they were derived [215]. Targeting the Ras/Raf/MEK/ERK and Ras/PI3K/PTEN/mTOR pathways could be very important in terms of CIC elimination.

### Involvement of the Ras/Raf/MEK/ERK and PI3K/PTEN/Akt/mTOR Pathways in Suppression Cellular Senescence and Premature Aging

The Ras/Raf/MEK/ERK and PI3K/PTEN/Akt/mTOR pathways play key roles in regulation of diverse processes ranging from: autophagy DNA damage responses, cellular senescence and aging [217-237] Treatment of cells induced to undergo senescence with MEK, PI3K and mTOR inhibitors will prevent the induction of cellular senescence and aging [219-221]. These experiments have led to innovative hypothesis that cellular senescence results from the hyperactivation of proliferative pathways. Drugs used to treat diabetes (*e.g.,* Metformin) or inhibit signal transduction pathways (*e.g.,* Raf, MEK, PI3K, mTOR inhibitors) can inhibit cellular proliferation and cellular aging [229-234]. Similar effects on the prevention of cellular senescence were observed with Resveratol, the active component contained in the skins of red grapes which was shown to also inhibit mTOR and cellular senescence [229, 230]. Additional studies have shown that the commonly-prescribed diabetes drug Metformin will also inhibit mTOR and prevent cellular aging [234]. Since both the Raf/MEK/ERK and PI3K/PTEN/Akt/mTOR pathways interact to regulate the activity of mTOR and downstream components of this pathway which are critical for both mRNA stability and protein translation, it is believed that by inhibiting some of these key pathways, it may be possible to prevent cellular aging (See Figures 1-3).

## CONCLUSIONS

Over the past 25 years, there has been significant progress in elucidating the involvement of the Ras/Raf/MEK/ERK and Ras/PI3K/PTEN/Akt/mTOR cascades in promoting cell growth, regulating apoptosis, chemotherapeutic drug resistance and more recently, cellular senescence and aging. Initial seminal studies performed in the late 70's and early 80's elucidated that oncogenes were present in the genomes of avian and murine retroviruses. Many of the viral oncogenes: *ErbB, Fms, Ras, PI3K, Akt, Src, Abl, Raf, Fos, Jun, Ets and NF-κB (Rel)* were subsequently identified as cellular genes which in some cases were captured by retroviruses. Now we know that these cellular genes are frequently abnormally regulated in human cancer. Furthermore mutations in human cancer often occur in upstream receptor genes such as *EGFR, HER2, Flt-2, PDGFR, Fms,* as well as chromosomal translocations (*e.g., BCR-ABL, TEL-PDGFR*) that serve to activate the Ras/Raf/MEK/ERK and Ras/PI3K/PTEN/Akt/mTOR pathways which have been discussed as playing critical roles in cellular proliferation in this review. Hence the Ras/Raf/MEK/ERK and Ras/PI3K/Akt/mTOR pathways are important therapeutic targets. Both of these pathways also interact with the p53 and Wnt pathways, which also play critical roles in regulation of cell growth, aging, CICs and metastasis. Specific Raf, MEK, PI3K, Akt, mTOR and Mdm-2 inhibitors have been developed and represent promising therapies for cancer and other proliferative diseases including premature aging.

Scientists and clinicians often have an intentionally narrow view of a particular topic. For example, cancer researchers predominately consider that Raf, MEK, PI3K, Akt and mTOR inhibitors will suppress the growth of malignant cancer cells. Yet MEK and mTOR and other inhibitors may also be useful in the treatment of diseases and disorders where there is abnormal cellular proliferation. Recent reports have also demonstrated that the suppression of the Ras/Raf/MEK/ERK and Ras/PI3K/PTEN/Akt/mTOR pathways may prevent the induction of cellular senescence and aging. Clearly, this later topic, aging, greatly enhances the potential clinical uses of these targeted therapeutic drugs. In conclusion, the Ras/Raf/MEK/ERK and Ras/PI3K/PTEN/Akt/mTOR pathways are prime therapeutic targets for diverse human diseases as well as aging.

Cancer therapy is often complex as there are relatively few cancers which proliferate in response to a single mutation preventing them from being treated with a mono-specific drug. One exception is the use of the drug Gleevec (Imatinb) for the treatment of chronic myeloid leukemia (CML). Although even with this therapeutic approach, resistance develops. Scientists and clinicians have developed newer BCR-ABL inhibitors (*e.g*., Dasatinib, Nilotinib, Bosutinib) which can reduce resistance which has also resulted in more through analysis and understanding of how the BCR-ABL kinase functions and resistance can arise by additional genetic mutations. These studies on BCR-ABL inhibitors have also paved the way for development of more effective inhibitors for other oncogenes.

It is possible that activation of the Ras/Raf/MEK/ERK and Ras/PI3K/PTEN/Akt/mTOR survival pathways by additional mutations in upstream oncogenes may replace the tumor's initial oncogene addition. This may complicate therapy as the tumor may no longer be responsive to treatment with a single inhibitor which targets the original oncogene responsible for malignant transformation as the cells now have additional downstream signalling pathways activated. In addition, the tumor cells may acquire subsequent mutations which make them resistant to inhibitors that target the original activated oncogene. Such mutations may occur in the original activated oncogene or in additional genes which are critical in anti-apoptotic survival cascades. These observations document the need for further elucidation of mechanisms of inhibitor resistance as well as the development of additional inhibitors which target either the mutated oncogene or other genes activated in the resistant cells.

The activation of multiple signalling pathways by many oncogenes illustrates the need for the targeting of more than one signalling pathway. Although one inhibitor which targets one molecule in one pathway may initially appear to be effective in inhibiting tumor cell growth, the cell may adapt and be able to survive due to the activation of an additional signalling pathway. Although the Ras/Raf/MEK/ERK and Ras/PI3K/PTEN/Akt/mTOR pathways have distinct effects on cell proliferation, they have many common downstream targets that may be able to function in promoting survival in the absence of the corresponding functional pathway. In some cases resistance to small molecule inhibitors may be due to the activation of an additional pathway that also serves to promote survival (*e.g., PIK3CA* and *HRAS* mutations can confer resistance to MEK inhibitors and other targeted therapeutics such as Erbitux and others) [238-245].

Most cancers are more complex and often the genes and events involved are either not known or difficult to counterbalance. Chemotherapy and radiotherapy can be effective in the treatment of certain tumors, however, often cancers become resistant to these approaches, perhaps due to the emergence of CICs [203, 215-217]. Thus scientists and clinicians have endeavored to develop more specific therapies that target key pathways involved in cancer growth. In this respect, the Ras/Raf/MEK/ERK and Ras/PI3K/PTEN/mTOR/Akt pathways represent key therapeutic targets as they are often dysregulated by various mutations in cancer and these cascades control the activities of many proteins critical for cell growth and metastasis. In fact, these pathways are already being targeted in certain cancer patients. However, usually the cancer patients being treated with inhibitors that target these cascades have diseases that often have poor prognoses. That being said, what are the pros and cons of targeting these pathways? Let us first consider the positive aspects of targeting these pathways. First, these pathways are frequently activated in human cancer, thus in many cases, targeting the cascades will suppress cell growth, in the absence of knowing the precise mutation(s) responsible for the cancer. Second, although the biochemical interactions of these pathways are quite complex, there is quite a bit of knowledge of how these pathways function. Third, some inhibitors which target key components in this pathway (*e.g*., rapamycin which targets mTOR) have undergone extensive evaluation in humans as they have been used to prevent allograft rejection in kidney and other transplant patients for many years. Fourth, targeting these pathways may prevent aging and cellular senescence.

Now, let us summarize some of the cons of targeting these pathways. First, an obvious problem results from these pathways controlling the expression of many downstream targets (easily in the 1000's), thus inhibiting these pathways will be detrimental in certain cells, unless it is possible to deliver the inhibitor to specifically the cancer cell. Second,the Ras/Raf/MEK/ERK and Ras/PI3K/PTEN/Akt/mTOR pathways cross regulate each other and other pathways including the Wnt/β-cateinin pathway which is critical for many aspects of cellular growth and differentiation including the EMT. The Ras/Raf/MEK/ERK and Ras/PI3K/PTEN/Akt/mTOR pathways also regulate other pathways which have not been discussed in this manuscript. These other pathways include: the Jak/STAT, NF-κB and transforming growth factor-β (TGF-β) pathways which can be directly and indirectly regulated by ERK and Akt phosphorylation [62]. In this regards there will be a Ying-Yang effect, when one cascade is inhibited, components of the other pathway could be deregulated. Third, inhibitors that target these pathways are often cytostatic and not cytotoxic, that is somewhat logical as if these inhibitors were cytotoxic, there would be massive toxicity problems. To get around this problem, inhibitors targeting these pathways could be combined with cytotoxic chemotherapeutic drugs or radiation therapy that affects the rapidly growing cancer cell. In summary, the Ras/Raf/MEK/ERK and Ras/PI3K/PTEN/Akt/mTOR cascades are complex, interacting pathways playing key roles in normal and malignant cell growth. These pathways are frequently activated by mutations in human cancer. They represent key therapeutic targets for cancer and various other diseases as well as the prevention of aging.
